# Theobald Smith

**DOI:** 10.3201/eid1412.081188

**Published:** 2008-12

**Authors:** Myron Schultz

**Affiliations:** Centers for Disease Control and Prevention, Atlanta, Georgia, USA

**Keywords:** Photo quiz

**Figure:**
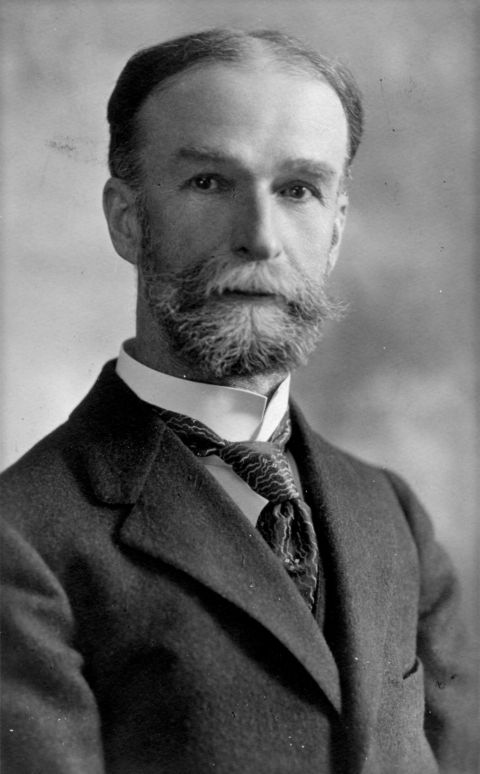
Theobald Smith

This is a photograph of Theobald Smith (1859–1934). Smith was a pioneer epidemiologist, bacteriologist, and pathologist who made many contributions to medical science that were of far-reaching importance. He is best known for his work on Texas cattle fever, in which he and his colleagues discovered the protozoan agent and its means of transmission by ticks. This was the first time that an arthropod had been definitively linked with the transmission of an infectious disease.

Theobald Smith was born in 1859. He was the son of a German immigrant, who kept a small tailoring shop in Albany, New York. At age 18, Smith earned a tuition-free scholarship to Cornell University. He graduated from Cornell in 1881 with a Bachelor of Philosophy degree, and he received his MD at Albany Medical College in 1883. Realizing that his 2 years of study had not prepared him for the practice of medicine, Smith returned to Cornell for graduate study. Smith’s mentor at Cornell, Professor Simon Gage, helped him secure his first job at the newly formed Bureau of Animal Industry (BAI) in Washington, DC. Smith also established a department of bacteriology at Columbian University (now George Washington University), where he taught from 1886 to 1895. This was the first department of bacteriology at a medical school in the United States.

When he went to Washington, Smith knew very little bacteriology. He had not been able go to Europe to study with men like Pasteur, Koch, or Virchow. Because he could read and speak German almost as well as English and he could read French easily, he was able to study the papers of these masters and teach himself. Within a year of his arrival in Washington, Smith introduced Koch’s methods. At this time, he also began his life-long work on tuberculosis. At a later time, he successfully challenged Koch’s concept that human and bovine tuberculosis were caused by the same organism.

Smith’s work at BAI was extremely productive. BAI was created within the Department of Agriculture in 1884, when efforts by the states to stem the rising tide of animal diseases proved inadequate. The major problems were hog cholera, bovine pleuropneumonia, Texas cattle fever, turkey blackhead, and bovine tuberculosis. During his first 2 years at BAI, Smith discovered a new species of bacteria (*Salmonella enterica*, formerly called S*almonella choleraesuis*), which he thought was the cause of hog cholera. It was later shown that hog cholera was in fact a viral infection and Smith’s bacillus was a constant but secondary invader. Although this genus of bacteria was discovered by Smith, Daniel E. Salmon, Smith’s chief, claimed credit for the discovery, and the genus *Salmonella* is named after him. In 1886, Smith, collaborating with Salmon, presented the first proof that killed bacteria could be used to induce active immunity in experimental animals. This established the basis for the later development of protective immunization for human bacterial enteric diseases such as typhoid and cholera. Smith was the first person to use the fermentation tube to study bacterial physiology and classification, especially focusing on the details for differentiating aerobes, facultative anaerobes, or anaerobes and on characterizing fresh isolates thought to belong to these groups.

A few years after beginning his work at BAI, Smith turned his attention to Texas cattle fever, a devastating disease that destroyed 90% of herds in some affected areas. It occurred in northern cattle that came in contact with cattle from Texas during cattle drives to stockyards in Kansas, Missouri, Iowa, and Illinois. It was a problem of great economic and political importance. Cattle ranchers had long held a vague but persistent impression that ticks were in some manner the cause of the disease. Smith had the good sense to listen to the cattle ranchers and formulate a hypothesis based on these impressions that he tested with searching experiments to subject it to scientific scrutiny. Some confusion exists about the part that Smith played in the Texas cattle fever discovery. Smith is widely cited as the sole person who discovered that ticks were the vectors of Texas cattle fever, when in fact, it was a collaborative effort of Smith with his colleagues, Fred L. Kilbourne and Cooper Curtice, both veterinarians. Smith never claimed this work as solely his own, even though popular accounts entirely credited him.

In 1889, Smith described little bodies in the erythrocytes of infected cattle; he later recognized (1891) them as protozoa, which he eventually named *Piroplasma bigeminum* (now called *Babesia bigemina*). Following this discovery, Smith and Kilbourne conducted experiments in which they placed southern cattle in pens with northern cattle. In some instances, ticks were left on the infected animals; in other enclosures, the ticks were removed. The researchers also kept native cattle in fields in which infected ticks had been left on the ground. These transmission experiments established beyond question the role of ticks (*Boophilus* spp.) as the carrier of this disease. Smith’s 301-page monograph about the laboratory and field experiments, BAI Bulletin No. One (1893), is regarded as one of the classics of medical literature. In these experiments, it was also demonstrated that the infection could pass in ticks from adults to nymphs, a new and extraordinary phenomenon of parasitism. This research was conducted by Curtice. Delineation of the tick’s life cycle soon paved the way for control of the disease by dipping cattle to kill the ticks.

The discovery by Smith et al. that insects can transmit disease represents one of the fundamental steps forward that altered the entire course of medical science and public health. It presaged the discovery in the next few years of the insect transmission of trypanosomiasis of cattle (nagana) in 1895 by David Bruce, malaria in 1897 by Ronald Ross, yellow fever in 1900 by Walter Reed and his colleagues, and typhus in 1909 by Charles Nicolle.

In 1895, Smith reported that blackhead, an economically devastating enterohepatitis of turkeys, was caused by a protozoan called *Amoeba meleagridis* (now *Histomonas meleagridis*). Later, while at the Rockefeller Institute for Medical Research, Smith resolved the puzzle of transmission by discovering that embryonated eggs of the intestinal roundworm *Heterakis papillosa* (now *Heterakis gallinae*) could transmit the amoebas. This mechanism of transmitting a protozoan remains unique in the annals of parasitology.

In 1895, Smith moved to Cambridge, Massachusetts, to accept a dual appointment, serving as professor of comparative pathology at Harvard University and director of the Massachusetts State Antitoxin and Vaccine Laboratory. As director of this laboratory, Smith undertook many practical and theoretical studies on the production of tetanus and diphtheria antitoxins. He was one of the first to demonstrate the production of immunity by killed cultures of disease organisms and to show that a mixture of diphtheria toxin and antitoxin confers immunity. Under Smith’s administration, diphtheria antitoxin production increased from 1,700 to 33,000 doses within 4 years. Human deaths were reduced from 25% to 11% during the first year. Smith concluded that during the first 7 years of production of the antitoxin in the state laboratory, 10,000 lives had been saved by its use. Among Smith’s many fundamental contributions to immunology, the most important was demonstrating that animals develop hypersensitivity to bacteria upon repeated injections. What is now called anaphylaxis was long known as the “Theobald Smith phenomenon.”

In 1915, Smith joined the Rockefeller Institute for Medical Research as director of the Department of Animal Pathology, in Princeton, New Jersey. He remained there until his retirement in 1929. One of Smith’s accomplishments during this period was to clearly establish the criteria to distinguish between types of tubercle bacilli that affect humans and bovines. While Robert Koch asserted that bovine bacilli could not invade the human body, Smith took the position (which Koch later accepted) that the bovine organism could infect humans but was not the usual source of human infection. The bovine and human tubercle bacilli differ in many ways in their pathogenicity for animals and humans.

Regarding Smith’s personality, he was known as a patient, tenacious researcher whose experiments were meticulously planned. Hans Zinsser describes him as “a simple person who had the qualities of unpretentious probity and an instinctive integrity.” Smith authored 305 scientific publications, addresses, and government reports, usually as sole author. The last of Smith’s publications was his scientific credo, a treatise on parasitism and disease in which he put into context the great mass of his individual findings. When he was 74, a dinner was given in his honor in Philadelphia. He made many sage remarks about the nature of research. Among them he said, “In general, a fact is worth more than theories in the long run. The theory stimulates, but the fact remains and becomes fertile. The fertility of a discovery is perhaps the surest measure of its survival value.”

Among those who knew him, Smith was considered to be one of the most notable figures in American medicine of his period. Smith received 12 honorary degrees from leading universities and 11 medals. Among them was the Copley Gold Medal of the Royal Society, regarded as one of the highest scientific awards in the world at that time.
